# Discovery and Validation of a Novel Neutrophil Activation Marker Associated with Obesity

**DOI:** 10.1038/s41598-019-39764-4

**Published:** 2019-03-05

**Authors:** Yue Pan, Jeong-Hyeon Choi, Huidong Shi, Liwen Zhang, Shaoyong Su, Xiaoling Wang

**Affiliations:** 1 Georgia Prevention Institute, Augusta University, Augusta, Gerogia USA; 2 Georgia Cancer Center, Augusta University, Augusta, Georgia USA; 3 Proteomic Shared Resources, Mass Spectrometry and Proteomics Facility, Ohio State University, Columbus, USA

## Abstract

Obesity is accompanied by low-grade systemic inflammation that etiologically contributes to obesity-induced cardiovascular disease (CVD). Growing evidence supports that neutrophil, the most abundant type of leukocytes in human, is most likely to be the target peripheral leukocyte subtype initiating the inflammatory cascade in obesity. However, few studies have systematically assessed the genome wide changes in neutrophils associated with obesity. In this study, a hypothesis-free OMIC approach (i.e. the discovery phase) and a target approach (i.e. the validation phase) were used to identify obesity related neutrophil activation markers and their roles on CVD risks. In the discovery phase, genome wide DNA methylation, RNA-sequencing and quantitative proteomics were obtained from purified neutrophils (12 obese *vs*. 12 lean). In the validation phase, gene expression levels of the promising genes from the OMIC platforms were measured in 81 obese cases *vs*. 83 lean controls, and the association between the expression levels and CVD risks were evaluated. Significant difference was found for one gene, alkaline phosphatase, liver/bone/kidney (*ALPL*), across 3 OMIC platforms. In the validation phase, the gene expression levels of *ALPL* in leukocytes were significantly higher in obese compared with lean subjects (p < 0.05). Within the obese population, we observed that *ALPL* expression level showed significantly positive association with CVD risk factors (p < 0.05) including systolic blood pressure, diastolic blood pressure, mean arterial pressure, carotid intima–media thickness and borderline significance with fasting insulin (p = 0.08). This study identified one novel marker *ALPL* of neutrophil activation in response to obesity and provided evidence that obesity induced change in *ALPL* expression was associated with CVD risk factors.

## Introduction

Neutrophils have long been considered as simple suicide killers of the innate immunity, with primary roles against extracellular pathogens and in acute inflammation. Recently, novel functions of these cells, including secretion of chemokines and cytokines^1^ and shaping adaptive immune response^2–6^, have been uncovered. There has been a renewed interest and great appreciation of the role of neutrophils in chronic inflammation such as obesity and its related cardiovascular disease (CVD) risks. A series of animal studies^7–9^ published recently discovered and recognized the importance of neutrophil infiltration in obesity induced immune dysfunction. For example, Talukdar *et al*.^9^ found that neutrophil was the first immune cell responding to inflammation and its infiltration into the adipose tissue can occur as early as 3 days of high fat feeding. As the first cells at the site of inflammation, neutrophils secrete cytokines, paving the way for recruitment and activation of other cell types. Depletion of neutrophils can reduce atherosclerotic lesions and result in improvement in insulin sensitivity in mice fed on a high-fat diet^7,10^. Therefore, early activation of neutrophils should be an important area to target with for obesity research. However, limited human studies have been conducted on neutrophil activations in obesity status with majority of them only exploring circulating concentrations of certain granule proteins such as myeloperoxidase, neutrophil elastase, and defensins^11–13^.

While neutrophil depletion can reduce cardiometabolic abnormalities in mice fed on a high-fat diet, depleting neutrophils is clearly not a choice in humans. In this regard, identification of obesity induced neutrophil activation markers serves as a prerequisite to develop targeted treatment. Neutrophil activation is a multifaceted process that involves many biological pathways and it is difficult to pre-decide a list of genes based on the current knowledge. Accordingly, we conducted a series of OMIC studies in purified neutrophils, including genome wide DNA methylation, RNA-sequencing and label-free quantitative proteomics, to discover obesity related neutrophil activation markers from a comprehensive and systematic view. We further followed up our findings in a replication phase. Our previous study pointed to the activation of innate immunity in obesity and especially the activation of neutrophils in obese African American (AA) males^14^. For this reason, the discovery phase was conducted in AA males. We further expanded the findings to AA females in the replication phase. The current study provided convincing evidence that neutrophil alkaline phosphatase could serve as a novel marker of neutrophil activation in response to obesity and its related cardiometabolic risks.

## Methods

### Subjects

All subjects were selected from the EpiGO (EpiGenetic basis of Obesity induced cardiovascular disease and type 2 diabetes) study, which was established in 2011 aiming to identify peripheral DNA methylation changes involved in the pathogenesis of obesity and its related co-morbidities. This study in total recruited 378 obese (BMI ≥ 95^th^ percentile for age and gender) and 411 lean controls (BMI ≤ 50^th^ percentile for age and gender), aged 14–20, of both African Americans (n = 552, 49.5% obese, 54.2% female) and European Americans (n = 237, 44.3% obese, 49.4% female). All participants were free of chronic or acute disease and not on daily prescription medication for treatment of diseases. All subjects were recruited from the southeastern United States from 2011–2015.

The discovery phase was conducted in the participants of 12 obese cases and 12 age-matched lean controls selected from the EpiGO study using the criteria: (1) AA males; (2) having neutrophil DNA, RNA and protein available; (3) obese cases having a BMI ≥ 99^th^ percentile for age and sex, and lean controls having BMI < 30^th^ percentile for age and sex. The validation phase was conducted in additional 81 obese AAs and 83 age-matched lean controls from the EpiGO study using the criteria: (1) AA males or females; (2) having leukocyte RNA available.

### Cardiometabolic traits

Height and weight were measured by standard methods using a wall-mounted stadiometer and a scale, respectively. BMI was calculated as weight/height^2^. BMI percentile was calculated according to their age, sex, height and weight. Mean arterial pressure (MAP), systolic blood pressure (SBP) and diastolic BP (DBP) were measured with Dinamap monitors, using an appropriately sized BP cuff placed on the subject’s right arm. BP measurements were taken at 11, 13, and 15 minutes, during a 15-minute supine relaxation period. The average of the last two readings was used to represent MAP, SBP and DBP values. Fasting glucose levels were measured using Ektachem DT II system (Johnson and Johnson Clinical Diagnostics, Rochester, NY, USA) and fasting insulin was assayed in duplicate by specific radioimmunoassay (Linco Research, Inc., St Charles, MO, USA)^15^. Fasting serum triglyceride (TG), total cholesterol (TC), low-density lipoprotein (LDLC), and high-density lipoprotein cholesterol (HDLC) were measured using clorimetric method on Sirrus analyzer (Stanbio Laboratory, Boerne, TX).

### Assessment of carotid intima media thickness (IMT)

The common carotid’s IMT was measured using Hewlett-Packard Sonos 5500 (Andover, MA) equipped with a 7.5 MHz linear array probe. Ten frames from both the left and right common carotid arteries were analyzed by an experienced sonographer. The mean IMT was defined as the average of the IMT measurements and the maximum IMT was defined as the largest IMT measurement from both carotid arteries.

### Discovery step: OMIC on neutrophils

#### Neutrophil isolation and its DNA, RNA, protein extraction

Neutrophils were collected using a rapid and high purity (≥96%) method developed by De *et al*.^16^. Neutrophil/RBC mixture was gathered using cell preparation tubes (BD Biosciences, San Jose, CA). Neutrophils were obtained after lysis of the RBC from the mixture. Pelleted neutrophils were either stored at −80 °C immediately for future protein extraction or dissolved in RNA protect Cell Reagent (QIAGEN, Inc.) and stored at −80 °C for future DNA and RNA extraction.

Neutrophil DNA was extracted using QIAamp DNA mini Kit (QIAGEN, Inc.) and neutrophil RNA was extracted using QIAamp RNA mini Kit (QIAGEN, Inc.). Total RNA integrity following extraction was tested using the Agilent Technologies 2100 Bio analyzer (Agilent, Inc.) with the requirement of RNA Integrity Number (RIN) value ≥ 8. One case sample having RNA amount <200 ng was excluded for further RNA sequencing. Neutrophil protein was extracted from the stored pellets and the extracted proteins were digested with trypsin with peptide concentrations measured by Nanodrop (Thermo Scientific, Inc.). The same case sample that did not have enough RNA and another case sample that failed to provide enough protein for the quantitative proteomics were excluded. In summary, DNA methylation data were obtained for all the 24 discovery samples, while RNA sequencing data and quantitative proteomics data were obtained from 23 (11 cases *vs*. 12 controls) and 22 (10 cases *vs*. 12 controls) samples respectively.

#### Genome-wide DNA methylation

Genome-wide DNA methylation data were obtained using Illumina Infinium Human Methylation 450 K Beadchip (Illumina Inc.). The Minfi package^17^ and CPACOR (incorporating Control Probe Adjustment and reduction of global CORrelation) package^18^ were used for initial quantification, data preprocessing and quality control. For the quality control steps, detectable probes were defined as probes with detection p value < 1 × 10^−16^ in more than 95% samples and detectable samples were defined as samples with more than 95% CpG sites having a detection p value < 1 × 10^−16^). Probes on X and Y chromosomes were excluded.

#### RNA sequencing

RNA sequencing includes library preparation, sequencing and sequence alignment. TruSeq RNA Sample Preparation Kit (Illumina, Inc.) was used for library preparation. For sequencing, the RNA-seq libraries were subjected to 2 × 50 bp paired-end sequencing on a HiSeq2500 instrument (Illumina, Inc.) following standard protocols. Tuxedo protocol was used for the RNA sequence alignment^19^.

#### Quantitative proteomics

Mass-spectrometry based label-free quantitative proteomics were used. Protein identification were performed using nano LC/MSMS on a Thermo Scientific orbitrap Fusion mass spectrometer equipped with an EASY-Spray™ Sources operated in positive ion mode. For the protein quantification, spectral counts which were obtained from the MultiSpec method^20^ were used. In the spectral counting approach, relative protein quantification is achieved by comparing the number of identified MS/MS spectra from the same protein in multiple datasets.

### Validation step: leukocyte RNA extraction and genome wide gene expression assays

Leukocytes were collected after plasma removal and red blood cell (RBC) lysis. Pelleted leukocytes were dissolved in RNA protect Cell Reagent (QIAGEN, Inc.) and stored at −80 °C immediately. RNA extraction for leukocytes was the same as it for neutrophils with the identical RNA quality control. Illumina HumanHT-12 v4 Expression BeadChip (Illumina, Inc.) was used to obtain genome-wide gene expression data. Initial quantification was achieved by the Genome-Studio Gene Expression Module (Illumina, Inc.), and data preprocessing and quality control were achieved using lumi package^21^. The quality control steps included detectable probes (probes with detection p value < 0.05 in more than 50% of the samples), log transformation, quartile normalization, and batch adjustment. After these steps, the *ALPL* gene expression levels were selected for further analysis.

### Statistical analyses

In the discovery stage, the analyses on obesity related epigenomic, transcriptomic and proteomic changes in neutrophils were conducted for each platform separately. For DNA methylation analysis, the R package Limma^22^ was used for the identification of differentially methylated CpG sites between obese cases and lean controls. For RNA-sequencing analysis, the R package DESeq2^23^ was conducted on count-based mRNA data for the identification of differential expressed genes between cases and controls. For the quantitative proteomics analysis, the R package DESeq2 was also used to rank differential protein expressions from the spectral counting data.

In the validation stage, linear regression was used to test whether the *ALPL* expression levels differ between the obese and the lean groups and whether the difference was dependent on gender (i.e. group*sex interaction). Linear regression was also used to test its associations with cardio-metabolic traits and subclinical measurements of CVD in the obese group, including BP, fasting glucose, fasting insulin, fasting lipid profiles, and carotid IMT. A p value < 0.05 was defined as significance.

## Results

Table 1 lists the general characteristics of the discovery panel by obesity status. Quantitative proteomics identified 854 proteins in peripheral neutrophils, which is comparable to previous studies^24,25^. Out of the 854 proteins, 622 proteins were identified in more than 50% of the samples and were taken forward for differential analysis between cases and controls. The top 10 signals were listed in Supplementary Table S1. RNA sequencing identified 30,565 mRNAs in peripheral neutrophils, of which, 21,632 mRNAs had ≥1 count in more than 50% of the samples and were taken forward for differential analysis between cases and controls. The top 20 signals were listed in Supplementary Table S2. Supplementary Table S3 listed the top 20 signals of the differentially methylated CpG sites between cases and controls.

**Table 1:** General characteristics of the subjects in the OMIC study.

	Lean	Obese	P value^a^
N	12	12	—
Age (years)	17.5 ± 1.3	17 ± 1.5	NS
Age range (years)	15.9–19.7	15.1–19.1	—
BMI (kg/m^2^)	18.5 ± 1.3	41.7 ± 5.1	<0.001
BMI range (kg/m^2^)	16.6–20.7	36.4–51.3	—
BMI-percentile (%)	12.4 ± 10.6	99.6 ± 0.2	<0.001
BMI-percentile range (%)	0.6–29.4	99.4–99.9	—
SBP (mm Hg)	118.9 ± 8.8	119.4 ± 15.2	NS
SBP-percentile (%)	45.3 ± 28.5	47.1 ± 36.5	NS
DBP (mm Hg)	65.0 ± 4.0	64.7 ± 5.4	NS
DBP-percentile (%)	31.8 ± 16.3	37.4 ± 20.1	NS
Insulin (µu/mL)	8.6 ± 3.1	24.7 ± 11.3	<0.001
Glucose (mg/dL)	93.0 ± 5.8	94.3 ± 8.8	NS
TG (mg/dL)	62.1 ± 22.5	97.8 ± 36.5	<0.01
TC (mg/dL)	136.8 ± 25.7	176.9 ± 32.6	<0.01
HDLC (mg/dL)	54.0 ± 8.7	51.8 ± 6.0	NS
LDLC (mg/dL)	116 ± 27.8	157.4 ± 32.2	<0.01

Data are means ± SD. ^a^P-value adjusted for age (if applicable). BMI, body mass index (Obese: BMI-percentile ≥ 95^th^). SBP, systolic blood pressure; DBP, diastolic blood pressure (Hypertension: SBP-percentile ≥ 95^th^ or DBP-percentile ≥ 95^th^); TG, triglycerides; TC, total cholesterol; HDLC, high-density lipoprotein cholesterol; LDLC, low-density lipoprotein cholesterol. According to the 2011 Expert Panel on Integrated Guidelines for Cardiovascular Health and Risk Reduction in Children and Adolescents^37^, dyslipidemia can be defined as a presence of ≥1 of the following levels (mg/dL): TC ≥ 200, LDLC ≥ 130, HDLC < 40, and TG ≥ 130. Normal range of glucose: 70–120 mg/dl.

Using the cut-off of p < 0.05 across all three platforms, *ALPL* gene was identified with its gene expression (p = 5.8 × 10^−5^) and protein level (p = 2.7 × 10^−3^) significantly increased and its DNA methylation level significantly decreased (p = 5.9 × 10^−4^) in obese cases in comparison with lean controls (Table 2). *ALPL* gene was then taken forward for validation in the replication cohort.

**Table 2:** Integration of proteomic, transcriptomic and epigenomic results. P value < 0.05.

Gene name	Transcriptomics		Proteomics		DNA Methylation		
	P	Direction*	P	Direction*	CpG site	P	Direction*
*ALPL*	0.000058	+	0.002671	+	cg18789685	0.009252	−
					cg24722348	0.015466	−
					cg08727996	0.033684	−
					cg01037895	0.000595	−
					cg06346857	0.040962	−

*Direction: +, up-regulated in obese cases compare with lean controls; −, down-regulated in obese cases compare with lean controls.

**Figure 1:**
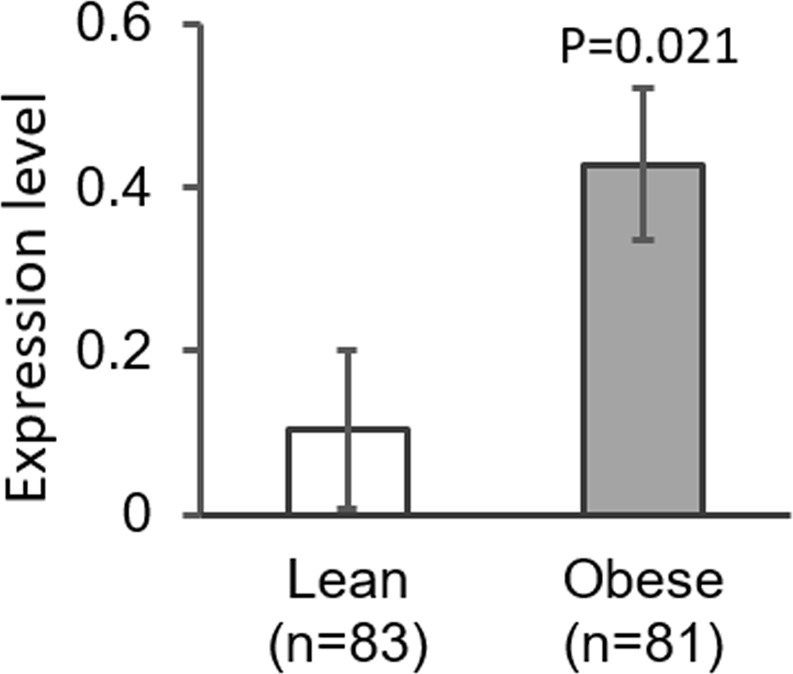
*ALPL* expression level between obese cases and lean controls (n = 164).

**Figure 2:**
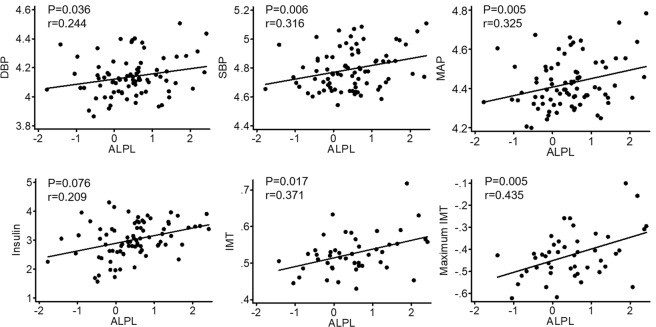
Correlations between *ALPL* expression level and CVD risk factors within obese population. The values of SBP, DBP, MAP, insulin, and maximum IMT were log-transformed.

Table 3 lists the general characteristics of the validation panel. The validation was conducted on gene expression level. Rather than using neutrophils, the validation was conducted on peripheral leukocytes, as in peripheral leukocytes, *ALPL* mRNA has only been observed in polymorphonuclear neutrophils^26^. As shown in Fig. 1, *ALPL* expression levels were significantly higher in obese cases in comparison with lean controls (p = 0.021) (Fig. 1). The group × gender interaction was not significant (p = 0.177), indicating that obesity associated higher expression of *ALPL* exits in both males and females. Within the obese group, we observed that *ALPL* expression level was significantly associated with SBP (p = 0.006), DBP (p = 0.036) and MAP (p = 0.005), with higher expression levels associated with higher BP levels (Fig. 2). We also observed that the *ALPL* expression level was positively associated with both the mean IMT (p = 0.017) and the maximum IMT (p = 0.005, Fig. 2). Additionally, the positive association between *ALPL* expression and fasting insulin reached borderline significance (p = 0.076, Fig. 2). Therefore, the gene expression analysis of a sample size of 164 confirmed that neutrophil *ALPL* had a higher expression level in obese cases compared with lean controls and its expression level was associated with cardiometabolic risk factors.

**Table 3:** General characteristics of the subjects in the validation phase.

	Lean	Obese	P-value^a^
N	83	81	—
Female (%)	50.6	54.3	NS
Age (years)	17.7 ± 1.7	17.8 ± 1.7	NS
Age range (years)	14.0–20.9	14.2–21.0	—
BMI (kg/m^2^)	18.8 ± 1.4	39.8 ± 7.1	<0.001
BMI range (kg/m^2^)	15.0–21.7	28.1–70.1	—
BMI-percentile (%)	19.1 ± 11.4	98.7 ± 1.1	<0.001
BMI-percentile range (%)	0–41.7	95.0–99.9	—
SBP (mm Hg)	107.3 ± 8.9	121.6 ± 17.4	<0.001
SBP-percentile (%)	28.3 ± 21.6	59.3 ± 33.7	<0.001
DBP (mm Hg)	64.1 ± 6.9	63.2 ± 8.7	NS
DBP-percentile (%)	39 ± 21.2	35.7 ± 24.6	NS
Insulin (µu/mL)	9.5 ± 5.1	24.3 ± 14.5	<0.001
Glucose (mg/dL)	86.8 ± 8.2	88.9 ± 7.9	NS
TG (mg/dL)	58.8 ± 20.6	68.9 ± 27.0	<0.01
TC (mg/dL)	151.5 ± 26.0	155.4 ± 32.9	NS
HDLC (mg/dL)	56.1 ± 11.9	44.0 ± 10.2	<0.001
LDLC (mg/dL)	83.7 ± 24.0	97.4 ± 30.6	<0.01
Mean IMT*	0.52 ± 0.05	0.53 ± 0.05	NS
Maximum IMT*	0.63 ± 0.06	0.65 ± 0.07	<0.05

*The numbers of subjects with carotid IMT measurements were 58 cases *vs*. 46 controls. Data are means ± SD. ^a^P-value adjusted for age (if applicable). BMI, body mass index. SBP, systolic blood pressure. DBP, diastolic blood pressure. TG, triglycerides. TC, total cholesterol. HDLC, high-density lipoprotein cholesterol. LDLC, low-density lipoprotein cholesterol.

## Discussion

In this study, we conducted a series of hypothesis-free OMIC studies of the epigenomic, transcriptomic and proteomic changes in neutrophils, from which a potential target gene, *ALPL*, was found to be more activated in obese subjects. We further replicated the expression change of *ALPL* in obese status, and observed the up-regulated *ALPL* expression level associated with CVD risk factors.

*ALPL* gene in neutrophils encodes neutrophil alkaline phosphatase (NAP)^27^, which is a membrane bounding glycosylated protein^28^ with its function of catalyzing dephospharylation and transphosphorylation reactions^29^. During bacterial infections, when the cells encounter stimulations of inflammatory signals, neutrophil NAP number dramatically increases and NAP-over-expressed neutrophils display enhanced chemotaxis, which promote its migration into inflammatory sites, ROS generation and apoptosis^30^. The role of NAP in chronic inflammation has not been explored. We for the first time showed that obesity was associated with NAP activation on the neutrophils and this activation was further linked with cardiovascular risks such as higher BP and higher carotid IMT, an acknowledged noninvasive marker for early atherosclerotic changes. We speculated that the activation of NAP in response to obesity per se or its associated obesogenic environment may promote neutrophils migration into atherosclerosis lesion site. While within the lesion site, NAP-over-expressed neutrophils may participate in the ROS generation, attract other inflammatory cells and enlarge the lesion site. Animal studies with specific deletion of NAP from neutrophils will be required to test this assumption.

Serum total alkaline phosphatase (ALP) level is commonly measured in clinic within the panel of liver function. However, this ALP measurement involves many *ALPL* encoded isoforms that named after their origin within the body, such as liver origin liver-ALP, bone-ALP, kidney-ALP, and other types. The subtypes differ only between post-translational modifications^31^. An interesting scenario is that although higher level of serum ALP has been consistently identified as an independent predictor for atherosclerosis and mortality of CVD in the general population, serum ALP levels are not associated with liver functions in the general population without evidence of liver disease^32^. Similarly, serum ALP levels were significantly higher in obese than in lean subjects^28^ but not linked with liver functions^33^. Although liver-ALP has been thought as the major contributor to serum ALP levels, one recent study^34^ observed a strong correlation between neutrophil counts and serum ALP indicating that the Increasing ALP may be originated from neutrophils. Incorporating with our observation that obesity is associated with higher transcription and translation of *ALPL* in neutrophils, directly study of serum NAP might provide an explanation for the serum ALP associated CVD risks.

The strength of the current study includes: (1) the two-step approach, which included a hypothesis-free OMIC step on purified neutrophils with 3 platforms as well as a replication step to validate the findings from the discovery phase and further establish the link between NAP and CVD risk factors; and (2) the focus on young-aged population at a pre-disease stage, allowing detection of obesity-specific neutrophil activation markers in the absence of co-morbidities and medication-related interactions.

Nevertheless, several limitations of this study need to be recognized. First, our protocol for neutrophil purification did not include a step to delete basophils and eosinophils that consist of about 3–5% of the granulocytes. De *et al*.^16^ showed that neutrophils isolated by this method have a purification of ≥96%. In a recent study^35^, this method was compared with StemCell untouched neutrophil isolation kit (negative selection of “highly pure” neutrophils) and observed that the negative selection did improve the purification (≥98%), but the overall yield of neutrophils was decreased by 50%. They also observed that low numbers (<5%) of contaminating basophils and eosinophils in neutrophil preparations contribute very little to the overall transcriptome profile of human neutrophils. Since neutrophil has limited amount of RNAs (i.e. 10–20 times less than monocytes), the yield of neutrophils is also an important factor to considerate in population studies. Second, this study was conducted in African American populations. Further replication in Caucasians as well as other ethnicities will be needed. It would also be interesting to examine the *ALPL* expression in different glucose tolerance groups. Third, studies have found that neutrophils from obese subjects are more responsive to chemotactic migration^36^, however, whether inhibition of NAP can reverse neutrophil migration activities has not been examined. Further research in this direction is warranted.

In conclusion, this study provided convincing evidence that NAP could serve as a novel marker of neutrophil activation in response to obesity and its associated CVD risks. Future studies of NAP may reveal more obesity induced inflammation pathways and contribute to the development of novel intervention strategies reducing the burden associated with obesity linked CVD risks.

### Ethics approval and consent to participate

This study was approved by the Institutional Review Board of Augusta University, and performed following the guidelines of the Declaration of Helsinki. Written informed consent was provided by all participants or by their parents if they were less than 18 years.

## Supplementary information

Supplementary Tables

## Data Availability

The data that support the findings of this study are available from the authors upon reasonable request and with permission of the Institutional Review of Augusta University.
