# Supplementary material for: Discovery and Validation of a Novel Neutrophil Activation Marker Associated with Obesity
Source: Sci Rep. 2019 Mar 5;9:3433. doi: 10.1038/s41598-019-39764-4 (PMC6400958; doi:10.1038/s41598-019-39764-4)
Supplement: Supplementary file 1 — Supplementary Tables [file 41598_2019_39764_MOESM1_ESM.pdf]

**Discovery and Validation of a Novel Neutrophil Activation Marker Associated with Obesity**

Yue Pan[1], Jeong-Hyeon Choi[2], Huidong Shi[2], Liwen Zhang[3], Shaoyong Su[1], Xiaoling Wang[1].

[1]Georgia Prevention Institute, Augusta University, Augusta, GA,

[2]Georgia Cancer Center, Augusta University, Augusta, GA,

[3]Proteomic Shared Resources, Mass Spectrometry and Proteomics Facility, Ohio State University, Columbus, Ohio.

Supplementary Table S1. Top 10 genes with their protein levels associated with obesity in purified neutrophils.

| Gene Name | P value | Spectral Counts | |
|---|---|---|---|
| | | Lean | Obese |
| ALPL | 0.001 | 15 | 51 |
| STT3B | 0.006 | 14 | 4 |
| VNN2 | 0.012 | 67 | 24 |
| C3 | 0.017 | 30 | 57 |
| MYL12B | 0.019 | 30 | 11 |
| YOD1 | 0.020 | 8 | 35 |
| PDCD6 | 0.021 | 13 | 25 |
| CD93 | 0.026 | 61 | 40 |
| PSMA4 | 0.028 | 17 | 47 |
| ASAH1 | 0.030 | 93 | 71 |

Supplementary Table S2. Top 20 genes with their expression levels associated with obesity in purified neutrophils.

| Gene Name | Fold Change* | P value |
|---|---|---|
| CASP5 | 2.46 | 2.00E-08 |
| TINF2 | 1.19 | 3.69E-08 |
| BCL2L11 | 1.84 | 1.48E-07 |
| SH3GLB1 | 1.58 | 4.85E-07 |
| WDR59 | 0.75 | 5.66E-07 |
| IFNAR1 | 1.26 | 1.73E-06 |
| PI4KA | 0.84 | 2.47E-06 |
| OAT | 2.51 | 3.51E-06 |
| DGKZ | 0.76 | 3.75E-06 |
| FFAR2 | 1.40 | 3.99E-06 |
| FAM129A | 1.51 | 5.50E-06 |
| PLEK | 1.30 | 8.12E-06 |
| UBXN4 | 1.17 | 8.24E-06 |
| TPR | 1.27 | 1.14E-05 |
| IL1B | 1.59 | 1.15E-05 |
| RBPJ | 1.28 | 1.33E-05 |
| DAPP1 | 1.32 | 1.36E-05 |
| TET2 | 1.31 | 1.37E-05 |
| LENG8 | 0.82 | 1.49E-05 |
| GK | 1.34 | 1.52E-05 |

Supplementary Table S3. Top 20 CpG sites with their methylation levels associated with obesity in purified neutrophils.

| CpG Site | Gene Name | Beta* | P value |
|---|---|---|---|
| cg27126872 | KBTBD11 | 0.0991251 | 0.0000486 |
| cg08269733 | CDH8 | -0.0349482 | 0.0000489 |
| cg24769628 | PCDHA6 | 0.0685704 | 0.000085 |
| cg10860308 | C10orf88 | 0.0434874 | 0.0000853 |
| cg05359853 | PPM1H | -0.0128915 | 0.0000864 |
| cg22582113 | FAM102A | -0.0186667 | 0.0000907 |
| cg06066676 | ATP10A | -0.0328985 | 0.0000957 |
| cg27467552 | PIM3 | -0.0509076 | 0.0001048 |
| cg16544956 | SP8 | -0.0114061 | 0.00011 |
| cg19214629 | CRLS1 | 0.0124844 | 0.0001256 |
| cg25154075 | MED13L | 0.0173008 | 0.0001299 |
| cg27338487 | GRP | 0.0128273 | 0.0001324 |
| cg26497348 | DSC3 | -0.0366982 | 0.0001335 |
| cg10008137 | GPX6 | -0.0244814 | 0.0001402 |
| cg02627403 | UNC13D | -0.0356996 | 0.0001466 |
| cg09233395 | TRPS1 | -0.0550059 | 0.0001502 |
| cg12195135 | ZNF584 | -0.0153043 | 0.0001538 |
| cg10182869 | TMEM196 | 0.0521222 | 0.0001731 |
| cg27209993 | GUCA1A | 0.0249388 | 0.0001777 |
| cg08180884 | ZNF536 | -0.0149752 | 0.0001935 |

*Beta value: the DNA methylation changes in cases in comparison with controls.
